# New-onset egg allergy in an adult: A case report

**DOI:** 10.3389/falgy.2024.1395807

**Published:** 2024-04-02

**Authors:** Michimasa Fujiwara, Takashi Kimura, Junya Ohira, Motohiro Inotani, Tomoko Sakane, Mizue Iwase, Sadanori Yamashita, Tooru Araki

**Affiliations:** Department of Pediatrics, NHO Fukuyama Medical Center, Fukuyama, Japan

**Keywords:** adult-onset, case report, egg allergy, ovalbumin, Gal d 2

## Abstract

Most adult cases of hen's egg allergy are carried over from childhood, and new-onset adult cases are rare. Such cases may result from cross-reactivity or sensitization by inhalation. Here we present a rare case of adult-onset egg allergy due to monosensitization to ovalbumin (Gal d 2) with an unclear sensitization pathway. A 27-year-old woman developed recurrent gastrointestinal symptoms after ingestion of raw and under-cooked eggs. She had never suffered from atopic dermatitis or food allergies. She had never kept birds as pets and had no history of exposure to egg allergens. Prick to prick testing was positive only with raw egg white. Specific IgE testing revealed monosensitization to Gal d 2. She was advised to avoid raw and undercooked eggs and her symptoms resolved. In the management of adult-onset egg allergy, evaluation of allergen components will lead to appropriate elimination guidelines, and investigation of sensitization pathways may help identify the cause of this disease.

## Introduction

Hen`s eggs are one of the most common causes of food allergies. Egg allergy primarily affects infants and young children (1). Most patients outgrow egg allergy by adolescence (2). Egg allergies in adults are rare and most are carryover from childhood. New-onset egg allergy in adults is unusual and usually develops as the “bird-egg syndrome” caused by cross-reactivity between serum albumins present in bird feathers and yolk (3) or as the “egg-egg syndrome” caused by occupational sensitization in workers in the bakery and confectionary industries (4). Since there are few reports of new-onset adult egg allergy due to causes other than these entities, its pathogenesis is not well understood. Here, we report a rare adult case of new-onset egg allergy due to ovalbumin monosensitization with unknown pathogenesis.

## Case report

A 27-year-old woman who was a clinical resident at our institution had recurrent episodes of nausea, abdominal pain, and diarrhea within an hour of ingesting various foods containing raw or undercooked eggs. The symptoms resolved within a few hours and there were no skin, mucous membrane, or respiratory complications. No symptoms were observed with cooked eggs. She had been able to consume chicken eggs since childhood without restriction. She also did not report any allergic disease such as atopic dermatitis. She had no history of oral lysozyme-containing medications, ovalbumin-containing vaccinations, accidental ovalbumin exposure by ocular injection or splashing, laboratory work with egg proteins in experiments, or previous blood infusions from egg allergic subjects. She had never kept birds as pets. Blood tests revealed specific IgE antibody levels (Immuno CAP; Thermo Fisher Scientific, Waltham, MA, USA) of 3.43 U/ml (class 3), 0.34 U/ml (class 0), 0.02 U/ml (class 0), 0.05 U/ml (class 0), and 0.02 U/ml (class 0) for egg white, yolk, ovomucoid (Gal d 1), parrot feathers, and parrot dander, respectively.

In addition, a skin-prick test was performed on the volar side of the forearm using a bifurcated needle (ALO bifurcated needle; TMI, Tokyo, Japan). The positive (10 mg/ml dihydrochloride histamine) and negative controls (0.9% saline) were 10 and 0 mm, respectively. A positive response was defined as wheal size equal to or greater than half the diameter of the positive control. Prick test with egg white extract yielded positive results (wheal diameter, 12 mm), whereas yolk extract yielded negative results. Prick-to-prick tests with raw egg white yielded positive results (wheal diameter, 11 mm), whereas boiled egg white and raw and boiled egg yolk yielded negative results (Table 1). Oral food challenge tests for both the whole hard-boiled yolk and egg white were negative, but the raw egg could not be administered because of lack of consent.

**Table 1:** Results of prick tests in a patient with suspected egg allergy.

	Wheal, mm	Erythema, mm
Control		
Histamin (positive)	10	23
Saline (negative)	0	2
Commercial extracts		
Egg white	12	22
Yolk	0	2
Prick-to-prick method		
Raw egg white	11	20
Raw yolk	0	3
Cooked egg white	0	3
Cooked yolk	0	3

The IgE assays for ovalbumin (Gal d 2), ovotransferrin (Gal d 3), and lysozyme (Gal d 4) were 4.62 U/ml (class 3), <0.01 U/ml (class 0), and <0.01 U/ml (class 0), respectively (obtained from Thermo Fisher Scientific).

Based on the clinical course and laboratory findings, we diagnosed the patient with adult-onset egg allergy. Since she was monosensitized to ovalbumin, we determined that the removal of well-cooked eggs was unnecessary. She reported no further episodes of immediate reactions since adhering to the avoidance of raw and undercooked eggs.

## Discussion

Here, we report a rare case of adult-onset egg allergy due to ovalbumin monosensitization with an unclear sensitization pathway. Measurement of allergenic components revealed that the patient was monosensitized to Gal d 2. Therefore, the elimination of eggs was limited to raw and undercooked eggs, which led to a minimal decrease in the patient's quality of life.

Recent evidence suggests potential disparities in the phenotypic presentation of IgE-mediated food allergy between childhood and adulthood (5). While previous studies have examined the natural progression of food allergy during childhood (6), limited studies have been conducted to investigate how oral immune tolerance to previously consumed foods is disrupted during adulthood. Also in present case, we could not explain a reason that might explain the disruption of oral tolerance constituted in childhood.

Egg proteins have been identified in both egg white and yolk (7). The main allergenic components of egg white are Gal d 1, Gal d 2, Gal d 3, and Gal d 4. Gal d 1 is the most allergenic egg glycoprotein, followed by Gal d 2, 3, and 4 (8). As Gal d 1 is heat-stable and Gal d 2 is heat-labile, most patients with monosensitization to Gal d 2 tolerate heated egg (9). In addition, IgE sensitization to Gal d 2 in the absence of IgE to Gal d 1 is considered a predictor of specific reactivity to raw egg (10). Our patient was monosensitized to Gal d 2 and tolerated heated eggs; therefore, we instructed to remove only raw and undercooked eggs. In adult-onset egg allergies, evaluation of allergen components, as in the present case, may lead to minimal food elimination.

Most reported cases of adult-onset egg allergy are caused by sensitization due to cross-reactivity or a clear pathway of sensitization. The bird-egg syndrome primarily manifests as respiratory symptoms upon exposure to birds and secondarily as allergic symptoms following the ingestion of eggs. This syndrome involves cross-sensitization to both yolk and various bird allergens, including feathers and droppings (3). This phenomenon underscores the cross-reactivity and sensitization via inhalation through the respiratory pathway. The egg-egg syndrome primarily manifests as respiratory symptoms upon exposure to egg aerosols in the bakery and confectionery industries and secondarily as allergic symptoms following the ingestion of eggs (4). This phenomenon also underscores sensitization through the respiratory pathway. In addition, adult-onset egg allergies due to transdermal sensitization via hand eczema (11) or due to sensitization to lysozymes caused by the oral administration of pharmaceutical preparations containing lysozyme have been reported (12). The present case did not fall into any of these categories. The history of exposure and the route of sensitization to Gal d 2 were not clarified in the detailed interviews.

In English literature, only four cases of new-onset egg allergy in adults with an unclear sensitization pathway have been reported so far (13–16). A review of these five cases, including the present case, is presented in Table 2. The ages were between 27 and 57 years, and there were four females and one male. In three of the five cases, the egg properties that induced allergic symptoms were limited to raw or undercooked eggs. Allergic symptoms varied from case to case, with two of the five cases presenting with anaphylaxis. Case 1 presented with symptoms localized to the mucosa and gastrointestinal tract. In addition, Case 4 and the present case presented with symptoms with localized gastrointestinal tract, suggesting the possibility of food protein-induced gastroenteritis syndrome. However, the results of specific IgE antibody and skin prick test were suggestive of IgE-mediated food allergy. The pattern of sensitization to eggs also differed from case to case, but Case 4 and the present case shared monosensitization to Gal d 2, and symptoms were induced only by raw or undercooked eggs in both cases. The cause of unusual clinical symptoms limited to gastrointestinal or mucosal symptoms lacking systemic reaction was considered to depend on sensitivity to enzymatic digestion (13) and the presence of intact gastrointestinal mucosa (16). In these cases, the pathogenesis of adult-onset egg allergy was unclear, with the possibility of stress noted only in Case 2. Prognosis was unclear in all cases. Because there is a lack of information on the pathogenesis and prognosis of adult-onset egg allergies, in which a clear pathway of sensitization cannot be identified, further case series are desirable.

**Table 2:** The review of adult onset egg allergy with an unknown pathogenesis.

Case	1	2	3	4	5
Age at onset	47	55	50	30	27
Sex	Female	Female	Female	Male	Female
Egg properties that induce symptoms	Raw or undercooked eggs	Egg or egg-containing products	Egg or egg-containing products	Raw or undercooked eggs	Raw or undercooked eggs
Allergic symptoms	Mucosal	Mucosal	Mucosal	Gastrointestinal	Gastrointestinal
	Gastrointestinal	Respiratory	Skin		
			Respiratory		
Anaphylaxis	-	+	+	-	-
Specific IgE					
EW (UA/ml)	N.D.	2.8	1.22	3.52	3.43
Yolk (UA/ml)	N.D.	Negative	0.44	N.D.	1.58
Ovomucoid (UA/ml)	N.D.	N.D.	N.D.	0.10	0.02
Ovalbumin (UA/ml)	N.D.	N.D.	N.D.	8.87	4.62
Skin prick test	EW (3+)	EW (+)	EW (+)	N.D.	EW (+)
	Yolk (3+)	Yolk (+)	Yolk (+)		Yolk (−)
		WE (+)	WE (+)		
Prick-to-prick test	N.D.		N.D.		Raw EW (+)
		Boiled EW (+)		Raw EW (+)	Raw Yolk (−)
		Boiled Yolk (−)		Boiled EW (+)	Boiled EW (−)
					Boiled Yolk (−)
Pathogenesis	Unclear	Stress	Unclear	Unclear	Unclear
Reference	(11)	(12)	(13)	(14)	Present case

EW, egg white; WE, whole egg; N.D., not described.

In conclusion, when managing adult-onset egg allergy, evaluation of allergen components will lead to appropriate removal guidance, and investigation of sensitization pathways may help identify the cause of this disease.

## Data Availability

The original contributions presented in the study are included in the article/Supplementary Material, further inquiries can be directed to the corresponding author.
